# Assessment of Lentiviral Vector Mediated CFTR Correction in Mice Using an Improved Rapid *in vivo* Nasal Potential Difference Measurement Protocol

**DOI:** 10.3389/fphar.2021.714452

**Published:** 2021-07-27

**Authors:** P. Cmielewski, J. Delhove, M. Donnelley, D. Parsons

**Affiliations:** ^1^ Respiratory and Sleep Medicine, Women’s and Children’s Hospital, Adelaide, SA, Australia; ^2^ Robinson Research Institute, University of Adelaide, Adelaide, SA, Australia; ^3^ Adelaide Medical School, University of Adelaide, Adelaide, SA, Australia

**Keywords:** gene therapy, lentival vector, potential difference measurement, CFTR, mouse models

## Abstract

Cystic Fibrosis (CF) is caused by a defect in the CF transmembrane conductance regulator (*CFTR*) gene responsible for epithelial ion transport. Nasal potential difference (PD) measurement is a well established diagnostic technique for assessing the efficacy of therapies in CF patients and animal models. The aim was to establish a rapid nasal PD protocol in mice and quantify the efficacy of lentiviral (LV) vector-based *CFTR* gene therapy. Anaesthetised wild-type (WT) and CF mice were non-surgically intubated and nasal PD measurements were made using a range of buffer flow rates. Addition of the cAMP agonist, isoproterenol, to the buffer sequence was then examined. The optimised rapid PD technique was then used to assess CFTR function produced by second and third generation LV-*CFTR* vectors. V5 epitope tagged-CFTR in nasal tissue was identified by immunohistochemistry. When intubated, mice tolerated higher flow rates. Isoproterenol could discriminate between WT and CF mice. Improved chloride transport was observed for the second and third generation LV-CFTR vectors, with up to 60% correction of the cAMP-driven chloride response towards WT. V5-CFTR was located in ciliated epithelial cells. The rapid PD technique enables improved functional assessment of the bioelectrical ion transport defect for both current and potential CF therapies.

## Introduction

Cystic fibrosis (CF) is one of the most common autosomal recessive genetic disorders in the Caucasian population, with the majority of the mortality and morbidity due to lung disease. CF is caused by a defect in the CF transmembrane conductance regulator (*CFTR*) gene, which results in abnormal CFTR protein levels. CFTR is classified as an adenosine triphosphate (ATP)-binding cassette transporter, and transports chloride (Cl^−^) and bicarbonate ions across the membranes of cells predominantly in the lungs, liver, pancreas, intestinal tract, reproductive tract and skin (Boucher, 2002; Davis, 2006; Quinton, 2008). The CFTR protein is primarily located on the apical surface of epithelial cell membranes, where the chloride ion channel transports salt and fluid across the epithelium to maintain surface hydration (i.e., transepithelial transport) (Riordan et al., 1989; Welsh et al., 1992).

CFTR electrolyte transport can be measured electrically as the transepithelial potential difference (PD). This technique involves using a sensitive high-impedance voltmeter to measure the voltage between a fluid-filled sensing electrode in the nose and a reference electrode placed elsewhere into the body (Knowles et al., 1981). The use of a fluid-filled electrode enables solutions that alter ion channel conductances to be introduced into the nasal cavity, and the resulting electrical responses related to specific ion channels can then be assessed.

As a consequence of the Cl^−^ defect in CF airways, a raised PD measurement can be used as a diagnostic tool (Middleton et al., 1994). The electrical characteristic of a CF airway is a greater negative (hyperpolarized) potential across the nasal airway at baseline, a large depolarization (more positive) in response to the sodium (Na^+^) channel blocker amiloride, and a reduced or absent response to the effect of a chloride-free environment compared to normal and heterozygous/carriers for CF (Knowles et al., 1981). There is also an impaired response to Cl^−^ stimulation in the presence of the β-adrenergic agonist isoproterenol (Iso) in the nasal airways of CF patients (Wilschanski et al., 2006; Bienvenu et al., 2010), a finding also noted in the lower airways in both paediatric and adult CF populations (Davies et al., 2005).

The ability to accurately measure the PD *in vivo* is particularly important when assessing therapies that alter CFTR, such as airway gene therapies that deliver a correct copy of *CFTR* to airway cells (De Boeck et al., 2013). To determine the effectiveness of a lentiviral (LV) gene therapy in live intact CF mice, we have used a well-established transepithelial PD technique in the nasal airways, originally based on the method described by Parsons et al. (2000), and subsequently used in a longitudinal study to measure the persistence of CFTR lentiviral airway gene therapy (Cmielewski et al., 2014). A range of other LV gene therapy studies for CF have utilised PD measurements as an outcome measure (Limberis et al., 2002; Stocker et al., 2009; Alton et al., 2017; Reyne et al., 2021).

The PD method identifies defects in the Cl^−^ transport levels across nasal epithelia by delivering a range of Krebs-Ringer Buffers (KRB) in a multi-step process beginning with normal basal KRB, and then sequentially adding amiloride and then low chloride (LC) KRB under amiloride. It can be used to assess the differences between normal and CF CFTR channel function by measuring Na^+^ hyperabsorption using baseline KRB and the response to the Na^+^ channel blocker, amiloride. An electrical gradient produced by perfusing LC KRB is required to assess Cl^−^ movement across the airways, and the change or lack of change in PD can be a determinant of CFTR function.

CFTR can also regulate other membrane proteins such as the up-regulation of an alternate outwardly rectifying Cl^−^ channel (Egan et al., 1992; Gabriel et al., 1993) and the inwardly rectifying renal outer medullary potassium channel (Hryciw and Guggino, 2000). The introduction of cyclic adenosine monophosphate (cAMP) agonists, ATP or CFTR inhibitors in the perfusion fluid sequence can aid in not only distinguishing between CF and non-CF, but also quantify the success of a functional CFTR treatment (Rowe et al., 2011). However, the use of an increased number of KRBs means that fluid delivery to a mouse’s nasal airways can accumulate both in the nasal passages and the lungs, disrupting nasal PD readings and causing respiratory difficulties. To attempt to mitigate these interferences we have previously used small volumes and slow flow rates. However, the result has been that each solution is slow to achieve a plateau, which typically takes up to 20 min per solution (e.g., three solutions can take up to 60 min). As a result, we cannot use as many perfusion choices as desired without dramatically increasing study time, and increasing the risk of adverse animal outcomes. Together, all of these issues can compromise the characterisation of channel function *in vivo*. An ideal system would allow more rapid perfusion flow rates to increase the number of KRB solutions that can be accommodated in a nasal PD measurement run, such as the β-agonist Iso, enabling assessment of CFTR function by cAMP-driven Cl^−^ transport without causing respiratory or anaesthesia issues.

This study had two objectives. Firstly, we examined whether the use of non-surgical intubation to maintain a patent airway throughout the PD procedure would allow higher flow rates and additional fluid perfusion solutions to be delivered, enabling improved characterisation. Secondly, we determined the ability to detect the functional effects of second and third generation LV-*CFTR* gene addition vectors in CF murine airways using this new rapid nasal PD protocol in mice.

## Methods

All animal studies were approved by the Animal Ethics Committee’s of the University of Adelaide (M-2015–100, M-2015–101, M-2017–083) and the Women’s and Children’s Health Network, Adelaide (AE1007 and AE1008). We first compared our standard nasal PD perfusion flow rate with increased flow rates in intubated wild-type (WT) mice to determine the optimum flow rate. That optimal flow rate was then applied to measuring the nasal PD in WT and CF mice using our standard KRB solutions. The addition of the β-agonist, Iso, in the KRB sequence was then examined in both WT and CF mice, using the newly optimised intubation assisted rapid PD method, to assess the benefits provided by this additional perfusion solution.

The new rapid PD method was next tested by assessing the success of a second generation nasal LV*-CFTR* gene addition treatment in CF mice to correct the electrophysiological defect and determine its repeatability. The efficacy of a more clinically-relevant third generation LV-*CFTR* vector with and without an epitope tag was also examined.

### Nasal Potential Difference

The PD measurement system was configured as previously described (Supplementary Figure 1) (Cmielewski et al., 2010; Cmielewski et al., 2014). Briefly, the end of a fine microloader pipette tip (Cat #5242 956.003, Eppendorf™, Germany) was inserted into one nostril of an anaesthetised mouse to a depth of 2.5–3 mm, and a fluid circuit was created by attaching the pipette tip to fine polyethylene tubing (PE-10, Cat # I-10338, SteriHealth, Australia) connected to agarose bridges (3% w/v in 0.9% NaCl), and to calomel electrodes (Hg_2_Cl_2_ in 3 M KCl, Cole-Parmer Instruments, United States). The reference needle-electrode was inserted subcutaneously into the abdomen of the mouse. The resulting electrical signal was displayed in millivolts (mV) using an Iso-Millivolt meter (World Precision Instruments, United States) and recorded using a PASPort Sensor (Pasco Scientific, United States) connected to a laptop using DataStudio Version 10 (Pasco Scientific, United States).

### Flow Rates

Normal C57Bl/6 wild-type (WT) and knock-out CF (*cftr* ^*(tm1unc)*^-Tg (FABp-CFTR)/1Jaw) mice (2–6 months of age) were anaesthetised with an intraperitoneal injection of a mix of medetomidine (1 mg/kg) and ketamine (75 mg/kg). To permit normal breathing during the increased rate of solution perfusion the mice were non-surgically intubated with an 20 G cannula (BD Insyte™ i. v. cannula) and fibre optic light as previously described (Cmielewski et al., 2017). Following all recovery procedures, anaesthesia was reversed using atipamezole (1 mg/kg), and mice were kept warm on a heating mat or in a pre-warmed incubator until fully recovered.

We first examined the feasibility of infusion rates of 10, 20 and 50 μl/min and compared these to our standard 1 μl/min in the same animal, using normal basal (B) and LC KRB to determine an optimal flow rate in WT mice (B, LC; *n* = 4). The sequence of infusion rates was performed in the same animal from 1 to 50 μl/min (*n* = 2) and in the reverse order from 50 to 1 μl/min (*n* = 2) to eliminate fluid bias.

The standard slow infusion rate (1 μl/min, non-intubated) was then compared to the optimal infusion rate (20 μl/min; see Results) in both WT and CF mice, using the addition of the Na^+^ channel blocker amiloride (A) and the cAMP driven Iso to the fluid sequence (B, B + A, LC + A; *n* = 9). However, the length of anaesthesia and fluid overload that occurred when using the 1 μl/min infusion rate resulted in rejection of the nasal PD recordings due to poor signal quality. Therefore the final solution of LC + A + Iso was only compared at the higher infusion rate group in which all mice were intubated (B, B + A, LC + A, LC + A + Iso; *n* = 5–9). When PD measurements were repeated in an animal, a minimum gap of one week was used between procedures to enable the airway epithelium to recover from any physical disturbances that may have occurred due to previous placement of the recording/perfusion cannula.

### Cloning of the V5 Epitope Tag Into the CCL-CFTR Vector

PCR amplification of the third generation lentiviral backbone, CCL-EF1α-CFTR, was performed using primers containing a Kozak sequence (bold), the V5 amino acid sequence (underlined), and a serine/glycine polypeptide linker (italics). The resulting vector contained an N-terminus V5-tagged CFTR whose expression was driven by the human elongation factor-alpha (EF1α) promoter. The V5 tag is used as a surrogate marker of CFTR as immunological detection of CFTR is generally very poor, whereas antibodies directed against the V5 epitope have high binding affinity and specificity (Table 1). The PCR parameters were: initial denaturation at 98°C for 40 s, followed by 18 cycles of 98°C for 20 s, 70–65°C (−0.3°C/cycle) for 20 s, 72°C for 5 min 30 s followed by a further 20 cycles of 98°C for 20 s, 65°C for 20 s, 72°C for 5 min 30 s, and a final elongation step of 72°C for 10 min. PCR product was fused using In-Fusion cloning to generate the pCCL-EF1α-V5-CFTR vector (designated CCL-V5-CFTR).

**TABLE 1:** Primer and V5 sequences used to PCR amplify the pCCL-EF1α-V5-CFTR vector.

V5-forward	ggatagcacc*agcggcggcggcggcagcggcggcagc*atgcagaggtcgcctctggaaaaggccag
V5-reverse	*cgccgccgc*tggtgctatccaggcccagcagcgggttcggaatcggtttgcc**catggtggc**ggtctctcacgacacctgaaatggaagaaaaaaactttgaaccactg

### Vector Production and Titering

Three LV-CFTR vectors pseudotyped with the vesicular stomatitis virus carrying the glycoprotein G (VSV-G) were produced by a range of large or small scale production methods, utilising the transient transfection of HEK293T cells, depending on experimental needs.

For the repeatability experiment, a second generation HIV-1 based (WT 5′ LTR) LV-CFTR under the EF1α promoter was produced by calcium phosphate coprecipitation in large-scale multilayer cell factors with a five plasmid trans-activating regulatory protein (Tat) dependent system, as previously described (Rout-Pitt et al., 2018). LV vector was diluted in FreeStyle™ 293 Expression Medium (Cat # 12338018, Life Technologies Corp, United States). The titre was determined by transducing NIH 3T3 cells, followed by quantitative real time PCR (CFX, BioRad, Australia) of proviral genomic DNA obtained after 4 weeks of culturing the transduced cells to dilute out potential contaminating plasmid DNA (Anson et al., 2007).

For the comparison experiments, small-scale production of third generation LV, either with or without a V5 tag using a CCL backbone (partially deleted 5′ LTR fused to CMV enhancer/promoter) under the EF1α promoter was performed as previously described (Buckley et al., 2015). Transcription in third generation vectors is initiated by the chimeric CMV promoter within the viral LTR (Dull et al., 1998). A deletion in the 3′LTR of the third generation vector abolishes the probability of full-length viral mRNA being produced and packaged following initial transduction (Zufferey et al., 1998), a significant safety feature that is currently required for any clinical vector. This four plasmid, tat-independent system consisted of 50 µg transfer plasmid, 32.5 µg pMDLg/RRE (gag-pol), 17.5 µg pMD2. G (VSV-G env), and 17.5 µgpRSV-Rev. LV vector was resuspended in OptiMem (Cat # 31985070, Life Technologies Corp, United States). Vectors were titered using a p24 ELISA kit (Cat # 080111, ZeptoMetrix, United States) as per manufacturer’s instructions using dilutions of 10^−8^.

### Immunocytochemistry for V5

The presence of the V5 tag in the third generation CFTR vector was validated in cell culture via immunocytochemistry (ICC) prior to delivery to animals. HEK293T cells were transduced with VSV-G pseudotyped CCL-V5-CFTR lentivirus. Samples were fixed in 4% paraformaldehyde for 10 min at room temperature before being washed in PBS. Cells were permeabilised by incubation in 1% bovine serum albumin (BSA) in PBS containing 0.1% Triton-X 100 for 10 min at room temperature. Cells were washed in PBS and blocked with 1% BSA in PBS for 1 h at room temperature. Samples were incubated in rabbit anti-V5 antibody (1:600) (Cat # 13,202, Cell Signalling Technologies, United States) diluted in 1% BSA + PBS +0.1% Tween-20 (PBST) overnight at 4°C. Samples were washed with 0.1% PBST before being incubated in donkey anti-rabbit Alexa Fluor 488 (1:600) (Cat # ab150073, Abcam, United Kingdom) diluted in 1% BSA + 0.1% PBST for 1 h at room temperature. Cells were washed in 0.1% PBST before incubation in DAPI for 5 min followed by rinsing in 0.1% PBST. The presence of V5 immunofluorescence was confirmed using a Nikon Eclipse Ts2 microscope with NIS-Elements imaging software Version 5.20.00.

### Nasal LV-CFTR Delivery

In all CFTR gene delivery studies, mice were anaesthetised as above and suspended by their front incisors and a single bolus of 4 µl 0.3% lysophosphatidylcholine in phosphate buffered saline (PBS) was pipetted into the right nostril. One hour later, 20 µl (2 × 10 µl aliquots) of LV-CFTR was delivered to the same nostril in the same manner. Anaesthesia was reversed, animals were allowed to recover, and the rapid PD method was tested at baseline (pre-LV delivery) and at least one week after LV-CFTR delivery.

A second generation, VSV-G pseudotyped LV vector expressing CFTR from the constitutively active EF1α physiological promoter was produced and resuspended in FreeStyle™ media and tested in CF mice (*n* = 9). The repeatability of this new rapid PD method was tested at baseline, one and two weeks after LV-CFTR delivery. We then examined the more clinically relevant CCL third generation LV vector resuspended in OptiMem. Vectors with and without a V5 epitope tag (*n* = 5) were tested to evaluate histological detection of the tag as a surrogate for CFTR presence. PD assessment was performed one week after nasal administration, and compared to baseline.

After the last PD measurement mice were humanely killed by carbon dioxide asphyxiation. Following decapitation the fur was removed and heads were placed into 10% formalin for at least 24 h, and the bone was decalcified with 25% EDTA at 37°C for 2–3 days. Samples were transferred to 70% ethanol and, once the lower jaw was removed, the heads were sliced into several standard locations (Mery et al., 1994; Kremer et al., 2007), paraffin embedded and 5 µm sections mounted onto 0.85% 3-aminopropyltriethoxysilane (Cat #A36487, Sigma-Merck, United States) coated glass microscope slides prior to V5 epitope immunostaining.

### Immunohistochemistry for V5

Sections of mouse nasal samples and archival rat lungs treated with CCL-V5-CFTR lentivirus as a positive control (McIntyre et al., 2018; Reyne et al., 2021) were deparaffinised by immersion in xylene twice for 10 min. Slides were brought to water through decreasing concentrations of ethanol with a final rinse in distilled water. Antigen retrieval was performed by heating slides in a 10 mM sodium citrate buffer at pH 6.0 until boiling followed by simmering for 20 min. Once cooled to room temperature, slides were rinsed in 0.05% PBST, permeabilised in 0.3% Triton-X 100 in PBS for 10 min and again rinsed with 0.05% PBST. Sections were blocked for 1 h at room temperature with 1% BSA in PBS and then incubated with goat anti-V5 primary antibody (1:300) (Cat # ab95038, Abcam, United Kingdom) diluted in 1% BSA + 0.1% PBST overnight at 4°C. Tissue sections were rinsed in 0.05% PBST and incubated in donkey anti-goat Alexa Fluor 568 (1:400) (Cat # ab175704, Abcam, United Kingdom) made in 1% BSA + 0.1% PBST for 1 h in the dark. Samples were washed with 0.05% PBST, counterstained with DAPI for approximately 10 min, washed again, and mounted with ProLong™ Diamond antifade mounting media (Cat #P36961, Life Technologies, United States). Negative controls of mouse nasal tissue that did not receive the CCL-V5-CFTR vector were included, as well as reagent only, and secondary only antibody controls. Slides were visualised for the presence of V5 under an Olympus BX51 research-grade optical microscope DIC with AnalySIS imaging software, with ciliated cells identified based on their distinct morphology.

### Statistics

Statistical analyses were performed using GraphPad Prism Version 8 (GraphPad Software Inc., United States) with statistical significance set at *p* = 0.05 and power ≥ 0.8. All data are expressed as mean ±  SEM, unless stated elsewhere. Standard paired or unpaired t-tests were performed between pre- and post-data, or when there were two treatment groups, respectively. Multiple treatment groups were analysed by one-way analysis of variance (ANOVA) or repeated measures (RM ANOVA) as appropriate, with post-test multiple comparisons. If data failed normality, non-parametric methods were used.

## Results

### Nasal Potential Difference Flow Rates

**FIGURE 1:**
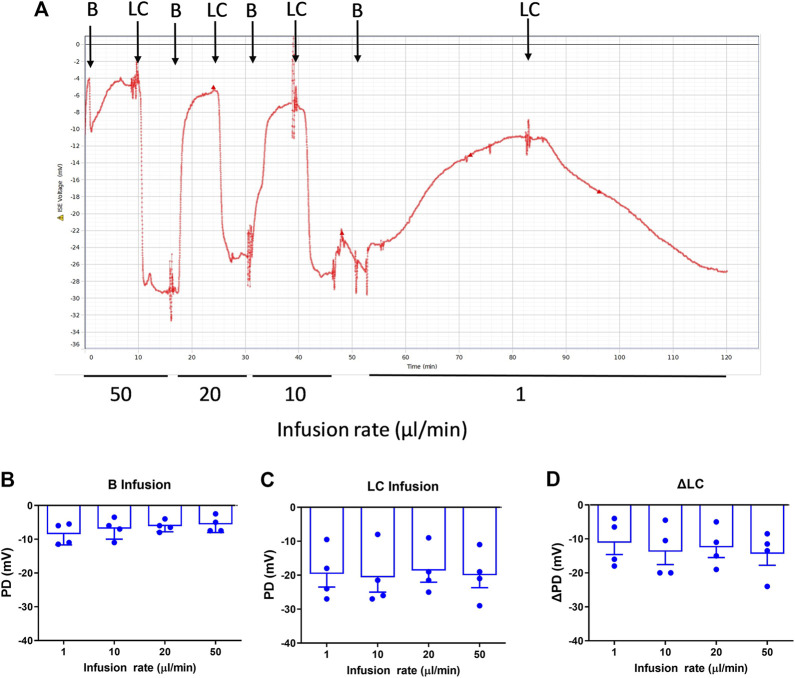
Nasal PD measurements at different infusion rates in WT mice. **(A)** Typical nasal PD trace showing the reproducibility of the rapid PD method (here, alternating basal and low chloride perfusions) in the same animal. The mean response following **(B)** Basal (B), **(C)** Low chloride (LC) KRB and **(D)** the difference between LC and Basal (ΔLC), n.s. Dunnett’s RM ANOVA, mean±SEM, *n* = 4.

When intubated, the mice tolerated the higher flow rates (10, 20, 50 μl/min) without any incidents. For the higher flow rates there was no significant difference in PD measurements (Figure 1) for Basal, LC KRB, or the change in nasal PD (ΔPD) in normal mice under LC conditions, when compared to our standard infusion rate of 1 μl/min (n.s., RM ANOVA, *n* = 4).

The optimal flow rate for rapid and consistently stabilised PD recordings was the 20 μl/min infusion rate, which resulted in a typical mean reduction in PD assessment time for each solution to reach a stable plateau averaging from 16.4 min/solution (range of 12–32 min), to 6.5 min/solution (range of 5–12 min), i.e., 2.5 times faster. This reduction in time allowed for more KRB solutions to be added in the sequence without health or welfare issues related to potential morbidities associated with lengthy anaesthesia.

**FIGURE 2:**
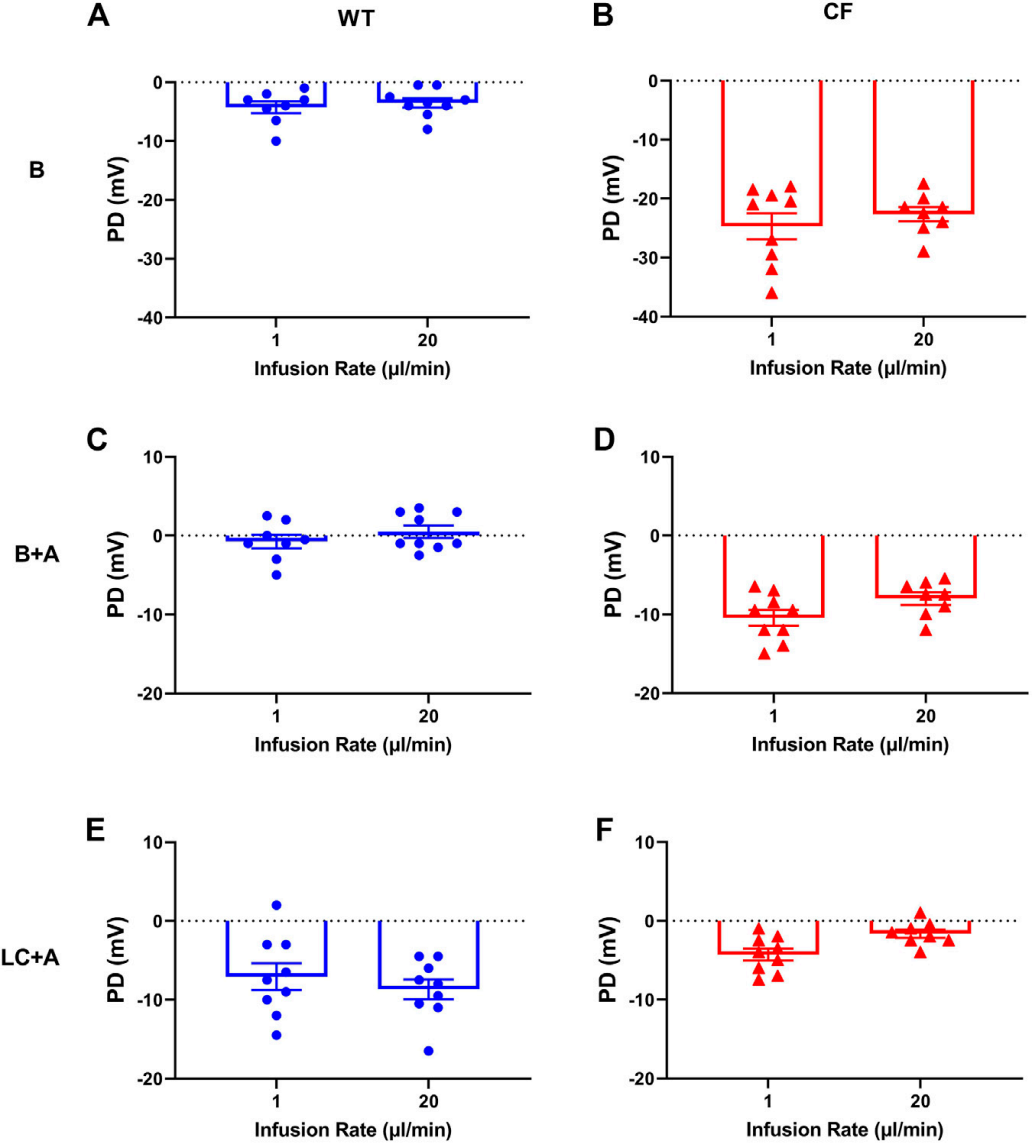
Characteristic nasal PD response of **(A,C,E)** WT (blue circle) and **(B,D,F)** CF mice (red triangle) after 1 and 20 μl/min performed 1 week apart following an infusion of basal KRB **(top panels)**, basal + amiloride (B + A) **(middle panels)** and Low chloride + amiloride (LC + A) **(bottom panels)**. No significant difference (n.s.) between 1 vs. 20 µl/min infusion rate, paired t-test, mean±SEM, *n* = 9).

**FIGURE 3:**
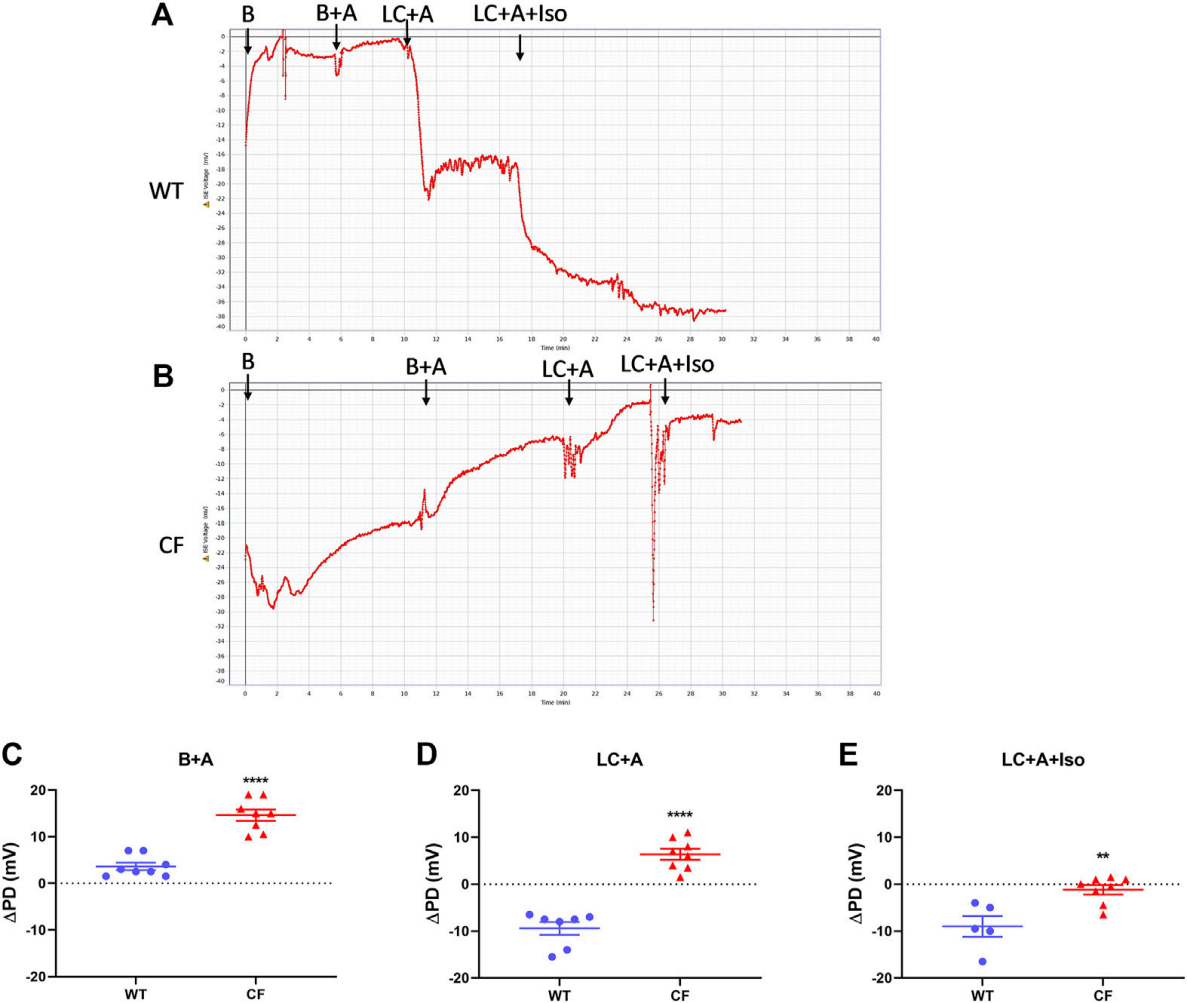
Nasal PD trace of **(A)** WT and **(B)** CF mice following infusion of KRB basal (B), basal + amiloride (B + A), Low chloride + amiloride (LC + A) and Low chloride + amiloride + isoproterenol (LC + A + Iso). ΔPD in WT (blue circle) and CF (red triangle) mice after **(C)** the addition of amiloride (B + A), **(D)** Low chloride (LC + A) and **(E)** the agonist isoproterenol (LC + A + Iso) into the sequence at the 20 μl/min infusion rate (**p < 0.01, ****p < 0.0001, t-test, mean±SEM, *n* = 5–9).

There was no significant difference in nasal PD when the 20 µl flow rate was compared to our standard 1 μl/min rate for both WT and CF mice with the addition of amiloride in the KRB sequence (Figure 2). When performed at the 20 µl flow rate the cAMP agonist isoproterenol could also be included in the KRB sequence for both WT (Figure 3A) and CF mice (Figure 3B) without any fluid overload issues or anaesthesia related morbidities. CF and WT mice displayed significantly different nasal ΔPD responses to the addition of amiloride in basal KRB (*p* < 0.0001; Figure 3C) and LC + A perfusion (*p* < 0.0001; Figure 3D). The addition of isoproterenol under the LC + A perfusion was also significantly different between CF and WT mice (*p* < 0.01; Figure 3E). These results demonstrate that the rapid method can characterise the altered nasal ion channel function in the CF animals.

### Nasal LV-CFTR Delivery

**FIGURE 4:**
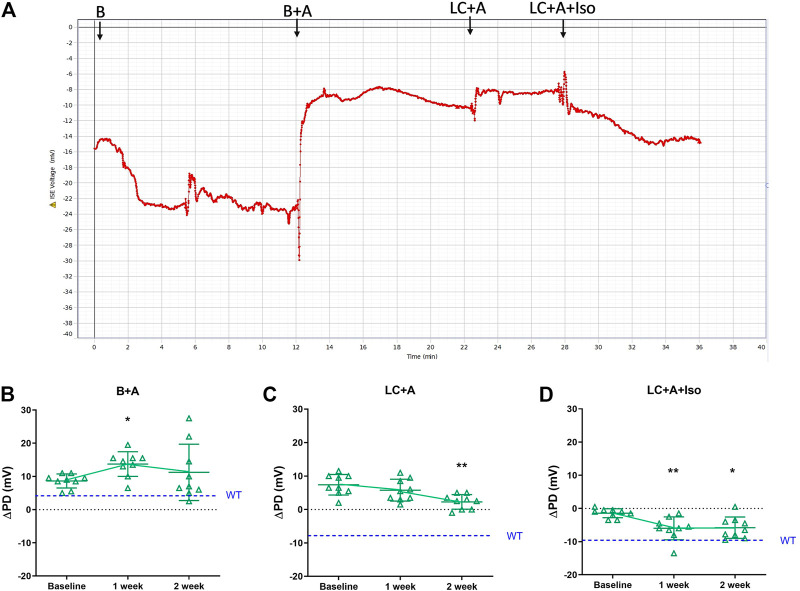
**(A)** Nasal PD trace of a CFTR treated CF mouse following infusion of KRB basal (B), basal + amiloride (B + A), Low chloride + amiloride (LC + A) and Low chloride + amiloride + isoproterenol (LC + A + Iso). ΔPD in CF mice before (Baseline), 1 and 2 weeks after second generation LV-*CFTR* gene addition to nasal airways following **(B)** Basal + Amiloride (B + A), **(C)** Low chloride under amiloride (LC + A) and **(D)** LC + A and isoproterenol (Iso) in KRB at the 20 μl/min infusion rate (*p < 0.05, **p < 0.01, Mean±SD, Dunnett’s RM ANOVA vs Baseline, *n* = 9). The ΔPD wildtype level (WT) designated by dashed blue line.

Following the successful introduction of the β-adrenergic agonist, isoproterenol, to the KRB sequence, we next tested if our new rapid PD technique could detect whether *CFTR* gene delivery corrected the electrophysiological defect in CF mouse nasal airways towards a more normal phenotype, and whether the measurements were repeatable. Second generation LV-*CFTR* vector was diluted in FreeStyle™ Medium, and delivered at a titre of 5.3 x10^8^ TU/ml. A typical nasal PD trace of a mouse that received LV-*CFTR* after 1 week is shown in Figure 4A. When compared to baseline there was a significant depolarisation in ΔPD response to amiloride one week after delivery of LV-*CFTR*, but this was not detected at week two (Figure 4B, *p* < 0.05, RM ANOVA, *n* = 9). In contrast, a significant hyperpolarisation (more negative response towards wildtype) to overall Cl^−^ transport was present after 2 weeks (Figure 4C, *p* < 0.01, RM ANOVA, *n* = 9), corresponding to an approximate 32% correction. Importantly, a significant hyperpolarisation to the isoproterenol cAMP driven Cl^−^ transport was present at both 1 and 2 weeks (Figure 4D, *p* < 0.01 and *p* < 0.05, respectively, RM ANOVA, *n* = 9), which corresponds to approximately 60% correction towards a normal wildtype bioelectrical functional response. These data suggest the chloride ion transport in CF mouse nasal airways was shifted towards a wildtype response following delivery of a second generation LV-*CFTR* vector.

### Third Generation LV-CFTR Vectors

To move towards a gene therapy for human CF airway disease a more clinically relevant LV vector was developed. Third generation CCL-*CFTR* vectors were constructed with and without a V5 tag (∼2.9 × 10^9^ vp/ml).

**FIGURE 5:**
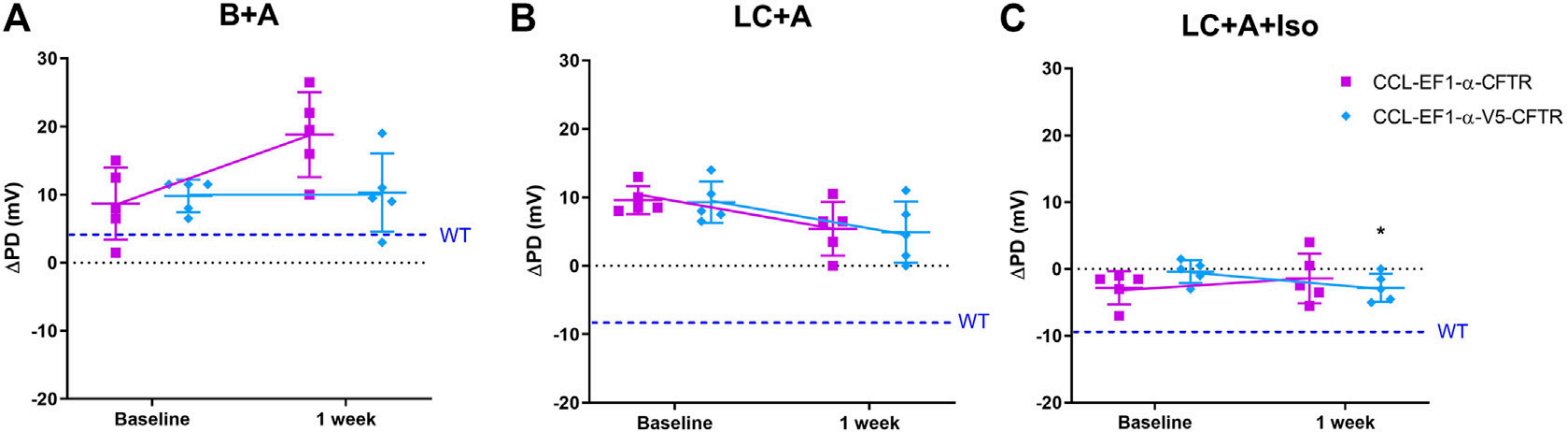
Electrophysiological responses (ΔPD) in CF mice before (baseline) and 1 week after delivery of a third generation CCL-*CFTR* (purple square), or CCL-*CFTR* with V5 tag (light blue diamond) vectors. Gene addition to nasal airways following **(A)** Basal + Amiloride (B + A), **(B)** Low chloride under amiloride (LC + A) and **(C)** LC + A and isoproterenol (Iso) in KRB at the 20 μl/min infusion rate (*p < 0.05, Mean±SD, paired t-test vs baseline, *n* = 5; n.s., unpaired t-test, CCL-*CFTR* vs CCL-V5-*CFTR*, *n* = 5). The ΔPD wildtype level (WT) designated by dashed blue line.

There was no difference in the ΔPD to the sodium channel blocker amiloride for all constructs 1 week after LV-*CFTR* delivery compared to baseline (Figure 5A) as expected. At one week, there was a hyperpolarisation of the ΔPD chloride response in the presence of amiloride however this did not reach significance for both third generation LV vectors (Figure 5B, n. s, paired t-test, *n* = 5).

In the mice that received the third generation CCL-V5-C*FTR* vector, but not the CCL-*CFTR* vector, the addition of the β-agonist Iso in the KRB sequence resulted in a significant change towards a normal bioelectrical response (Figure 5C, *p* < 0.05, paired t-test, *n* = 5). Furthermore there was no significant difference between the CCL-*CFTR* and CCL-V5-*CFTR* versions (n. s, unpaired t-test, *n* = 5), suggesting that the V5 epitope does not compromise exogenous CFTR functionality.

### Immunocytochemistry and Immunohistochemistry Validation and Detection of V5

**FIGURE 6:**
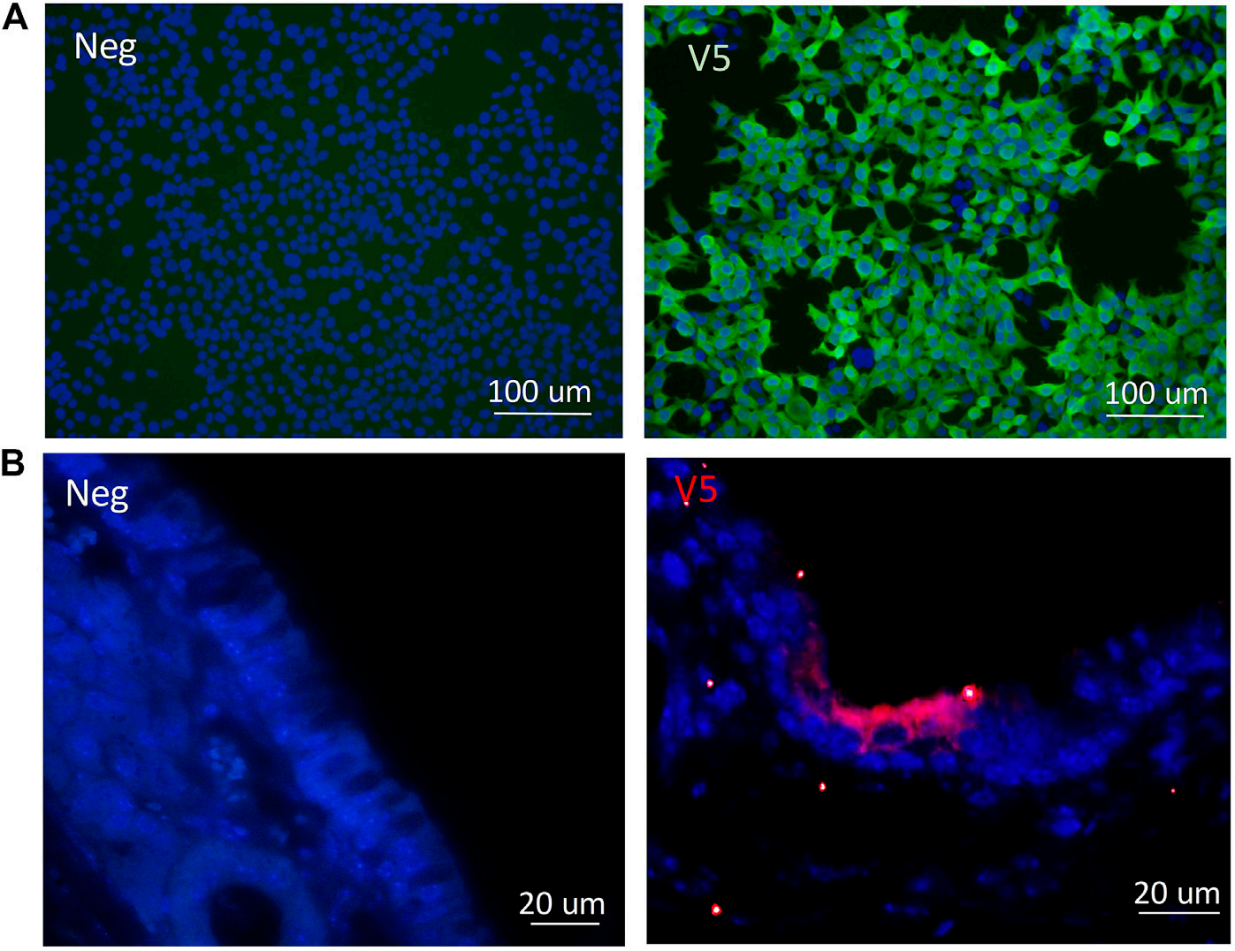
Validation of the V5 tag following delivery of the CCL-V5-*CFTR* vector in **(A)** *in vitro* samples of HEK293T cells by ICC; **(B)** in mouse ciliated nasal epithelium using IHC. All merged images (DAPI blue, V5 green or red) with non-CFTR treated cells or CF animals as negative controls (neg) on left panels, V5 staining images on right panels.

Following cloning of the V5 epitope tag into the CCL-*CFTR* lentiviral vector, detection of the V5 tag was performed by ICC. HEK293T cells were transduced with the modified V5-containing lentiviral vector, and the V5 tag was clearly detected using a V5-specific antibody (Figure 6A). V5 was detected in ciliated cells in nasal histological sections in mice that received the third generation CCL-V5-*CFTR* vector, validating its suitability to be used as a surrogate marker for CFTR protein detection (Figure 6B). Archival samples of rat lung transduced with the same vector were used as a positive control for immunohistochemistry (IHC), as they contained V5 staining within several bronchioles (Supplementary Figure 2). There was no V5 detected in any negative control samples from untreated CF animals.

## Discussion

In this paper we describe a rapid nasal PD method in a CF mouse model, in conjunction with non-surgical lung intubation, which provides greatly improved nasal PD measurement capability compared to previously described techniques (Cmielewski et al., 2014). Use of this approach eliminated potential adverse effects due to fluid accumulation in the lungs, and improved animal PD assessment throughput four-fold.

In other studies a slow rate of fluid perfusion, in conjunction with intubation assisted by oxygen supplementation with the mouse at a 15° head down tilt angle, has shown to be successful, however their approach was limited due to anaesthesia constraints (MacDonald et al., 2008). Other researchers have also used buffer solutions containing compounds that cannot be used clinically, low buffer infusion rates that could restrict their timing/recording of solutions, and different anaesthetics and body orientations (da Cunha et al., 2016). In contrast, in the present study we ensured that all infusions came to a steady-state plateau for accurate PD measurement, and the faster infusion rate allowed for the overall anaesthesia time to be reduced.

The faster infusion rate also allowed for the inclusion of additional perfusion solutions to further discriminate specific changes in the bioelectrical defect responsible for CF. Use of the β-agonist isoproterenol in the perfusion sequence allowed for measurement of cAMP directed chloride transport. Isoproterenol KRB is a clinically relevant perfusion used for phenotyping CF patients and for measuring the success of new treatments such as CFTR modulators (Pranke et al., 2017). Our rapid PD technique was also highly reproducible, enabling the same animal to have multiple assessments over time, which is a prerequisite for testing and monitoring CFTR therapies such as gene or cell addition, new CFTR modulators, or new drug treatments. Together, all of our changes have improved the quality and speed of nasal PD assessment.

We also demonstrated successful gene correction of the electrophysiological bioelectrical defect in the nasal airways of CF mice using a LV-CFTR under the non-viral human physiological EF1α promoter. Although this new vector was applied at approximately 40x lower titre than our previous LV-CFTR vector driven by the simian virus 40 promoter (Cmielewski et al., 2014), the level of correction of the electrophysiological defect observed here could provide a therapeutic benefit. Importantly, the third generation lentiviral vector–with lower titre, non-viral internal promoter and improved safety profile–provided not only correction of the chloride defect in murine nasal airways, but is a more clinically relevant vector that may be useful for future human CFTR gene therapy trials (White et al., 2017; Marquez Loza et al., 2019).

We have confirmed for the first time in mice that the N-terminus epitope tagging with V5 did not affect CFTR function, and was used to successfully detect CFTR expressing cells in the desired airway regions, i.e., in mouse ciliated nasal epithelium. Third generation batch titres were slightly higher than the second generation, but this may be a consequence of the titering assay. The method of detection of viral titre for the third generation vector was based on total p24 antigen concentrations, which overestimates non-functional empty viral particles compared to the real-time PCR technique used for the second generation vector that quantifies functional vector particles. These newer vectors are now ready for upscaled production (McCarron et al., 2016) for use in larger rodents and other CF models.

A limitation of this study was the use of a smaller cohort of mice for the third generation LV vector validation experiments, which likely reduced the study power. To adapt this rapid PD method into larger CF species, such as the CF rat (Tuggle et al., 2014; McCarron et al., 2020) or other CF models, the coupling of non-surgical intubation with the faster infusion of greater fluid volumes must first be optimised to ensure it is well tolerated. This optimisation will likely differ between species.

Together these data demonstrate an advance in both LV vector development and *in vivo* techniques for the detection and functional assessment of the bioelectrical ion transport defect for current and potential CF therapies.

## Data Availability

The original contributions presented in the study are included in the article/Supplementary Material, further inquiries can be directed to the corresponding author.
