# Supplementary material for: Assessment of Lentiviral Vector Mediated CFTR Correction in Mice Using an Improved Rapid in vivo Nasal Potential Difference Measurement Protocol
Source: Front Pharmacol. 2021 Jul 27;12:714452. doi: 10.3389/fphar.2021.714452 (PMC8353152; doi:10.3389/fphar.2021.714452)
Supplement: Supplementary file 1 [file Image2.pdf]

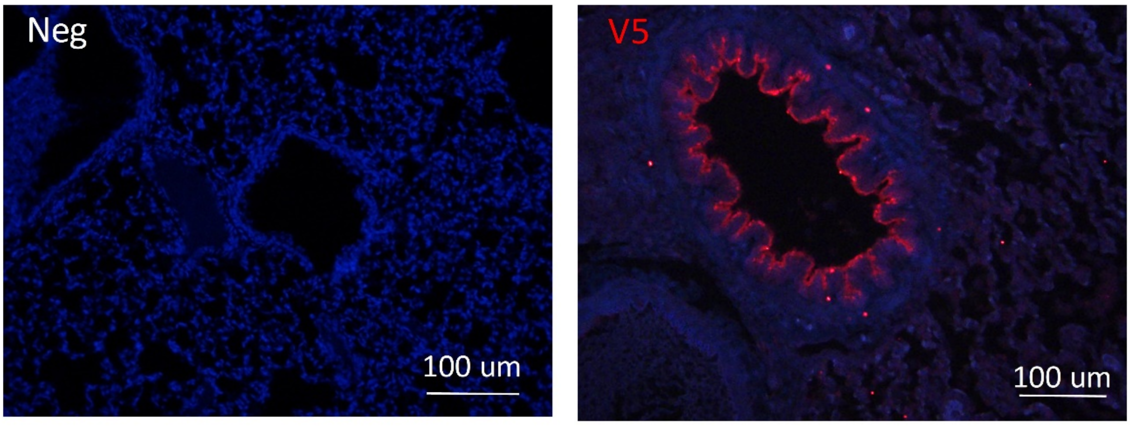

Supplementary Figure 2. IHC Controls

Archival negative (Neg) and positive (V5) controls in rat lung bronchioles using IHC following delivery of CCL-V5-*CFTR* to rat lungs. All merged images (DAPI blue, V5 red).
