# Supplementary material for: Assessment of Lentiviral Vector Mediated CFTR Correction in Mice Using an Improved Rapid in vivo Nasal Potential Difference Measurement Protocol
Source: Front Pharmacol. 2021 Jul 27;12:714452. doi: 10.3389/fphar.2021.714452 (PMC8353152; doi:10.3389/fphar.2021.714452)
Supplement: Supplementary file 2 [file Image1.pdf]

a

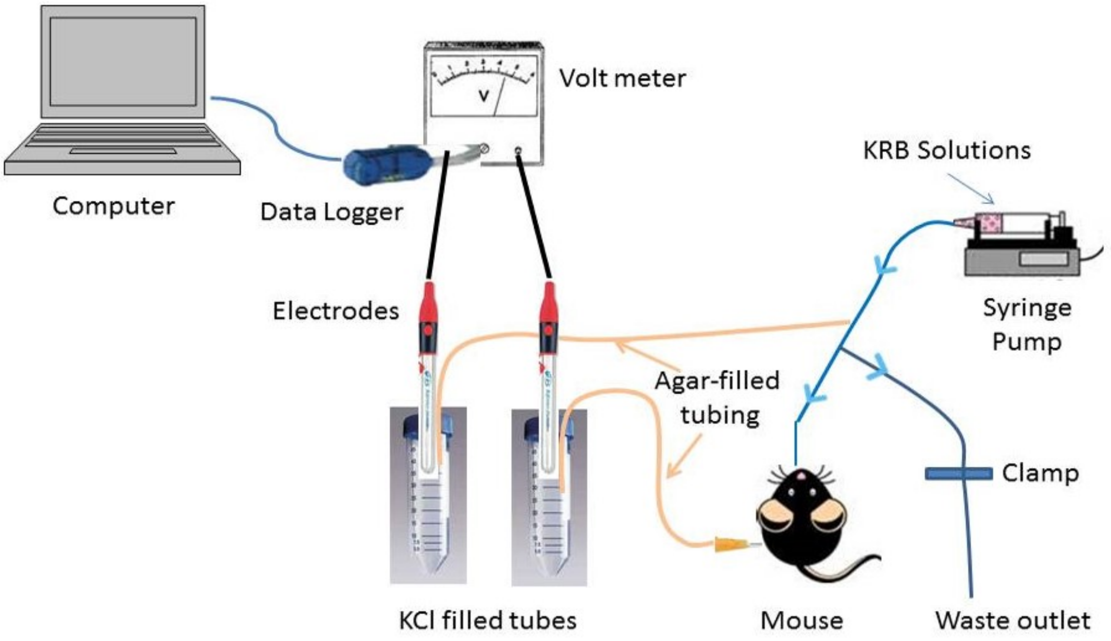

b

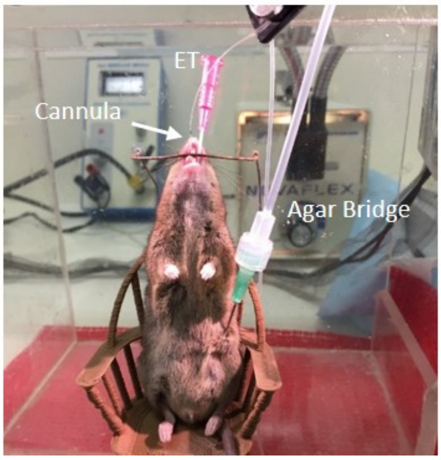

Supplementary Figure 1. Nasal PD Technique

a) A schematic diagram of nasal PD set up, and b) an intubated CF mouse with an endotracheal tube (ET) to assist in higher infusion rates during the nasal PD measurement. Fine cannula (arrow) filled with KRB inserted into the right nostril and an agar filled bridge inserted subcutaneous into the abdomen to complete the electrical circuit.
